# Corrosion Resistance of the CpTi G2 Cellular Lattice with TPMS Architecture for Gas Diffusion Electrodes

**DOI:** 10.3390/ma14010081

**Published:** 2020-12-26

**Authors:** Bożena Łosiewicz, Joanna Maszybrocka, Julian Kubisztal, Grzegorz Skrabalak, Andrzej Stwora

**Affiliations:** 1 Faculty of Science and Technology, Institute of Materials Engineering, University of Silesia in Katowice, 75 Pułku Piechoty 1A, 41-500 Chorzów, Poland; joanna.maszybrocka@us.edu.pl (J.M.); julian.kubisztal@us.edu.pl (J.K.); 2 Institute of Advanced Manufacturing Technology, Wrocławska 37A, 30-011 Kraków, Poland; grzegorz.skrabalak@ios.krakow.pl (G.S.); andrzej.stwora@ios.krakow.pl (A.S.)

**Keywords:** 3D cellular lattice structure, corrosion resistance, gas diffusion, aqueous metal-air batteries, selective laser melting, titanium, triply periodic minimal surface

## Abstract

The corrosion of materials used in the design of metal-air batteries may shorten their cycle life. Therefore, metal-based materials with enhanced electrochemical stability have attracted much attention. The purpose of this work was to determine the corrosion resistance of commercially pure titanium Grade 2 (CpTi G2) cellular lattice with the triply periodic minimal surfaces (TPMS) architecture of G80, D80, I-2Y80 in 0.1 M KOH solution saturated with oxygen at 25 °C. To produce CpTi G2 cellular lattices, selective laser melting technology was used which allowed us to obtain 3D cellular lattice structures with a controlled total porosity of 80%. For comparison, the bulk electrode was also investigated. SEM examination and statistical analysis of the surface topography maps of the CpTi G2 cellular lattices with the TPMS architecture revealed much more complex surface morphology compared to the bulk CpTi SLM. Corrosion resistance tests of the obtained materials were conducted using open circuit potential method, Tafel curves, anodic polarization curves, and electrochemical impedance spectroscopy. The highest corrosion resistance and the lowest material consumption per year were revealed for the CpTi G2 cellular lattice with TPMS architecture of G80, which can be proposed as promising material with increased corrosion resistance for gas diffusion in alkaline metal-air batteries.

## 1. Introduction

Metal-air batteries (MABs) are of great interest due to their high theoretical energy density and because they can be used in stationary, mobile, and electronic applications [1,2,3,4,5,6,7]. In the group of non-aqueous MABs, we can distinguish Li-air battery with the highest theoretical energy density, Na-air, and K-air batteries [7]. Aqueous MBAs include Fe-air, Zn-air, Mg-air, and Al-air batteries. In this group, the most promising for industrial scale applications are Zn-air and Al-air batteries with water-stable metal electrodes [5,6,8,9,10]. So far, only the Zn-air main battery has been successfully implemented on a mass scale. The aqueous MABs are especially attractive due to high energy density obtained as a result of eliminating the bulkier cathode chamber, high operation safety, high ionic conductivity, low cost, and environmental friendliness. From an economic point of view, abundant raw materials are also important. However, the Coulombic efficiency of the MABs is still too low for applications in electric vehicles and electronic devices. To overcome this problem, the optimizations of electrodes, electrolytes, and separator materials are necessary. Further efforts need to be made to increase the efficiency of both reduction and evolution reactions of oxygen, which require the development of long-term and effective bifunctional electrocatalysts in aqueous electrolytes.

MABs use porous gas diffusion electrodes (GDEs) for a sufficient supply of oxygen during discharge. The GDEs for use in alkaline fuel cells were already investigated in the 1960s. Many efforts have been made since then to optimize the design of the GDEs based on both experimental studies and computer simulations [1,2,3,4,5,6,8,9,10,11]. One of the most important factors that helps improve the performance of the GDEs in terms of their cycle life, capacity, and Coulombic efficiency using a static electrolyte, is an increase in the surface area of the GDEs and current collector. In the case of the most commonly used zinc electrode, its high surface area allows to lower the overpotential of zinc electrodeposition, which affects the elimination of undesirable dendrites formed during loading and the reduction of zinc passivation potential [12]. Also, consider that the hydrogen evolution reaction rate on the zinc electrode surface will increase with the surface development, which can lead to increased self-discharge of the battery when it is out of service, and lower Coulombic efficiency while charging the battery. Therefore, the aspect of hydrogen evolution reduction methods in the case of zinc electrodes with high surface area requires further research. An improvement of mechanical strength of the electrodes by minimizing their shape change is achieved through application of polymeric binders to bind the active powders. PTFE is the most commonly used [13]. However, this non-conductive polymer increases the electrode resistance which can limit zinc utilization. In order to reduce the resistance of zinc electrodes, carbon-based additives characterizing by high conductivity and good chemical resistance to alkaline solutions are used [14]. Their presence allows to avoid passivation helping to improve zinc utilization. The additives used in zinc electrodes also include heavy metals as Bi [15], In [16], Pb [13], Cd [17], Ti [18], and Sn [19]. These metals are characterized by higher reduction potentials in comparison with Zn and increase the conductivity of the Zn electrodes and current distribution as well. To prevent shape change of the zinc electrodes different solutions are proposed using coatings preventing the migration of the discharge product in the form of ${\mathrm{Zn}(\mathrm{OH})}_{4}^{2-}$ or discharge-trapping additives as Ca forming a solid compound of Ca(OH)_2_·2Zn(OH)_2_·2H_2_O with lower solubility as compared to ZnO in KOH solutions [6]. It was also reported in literature that suitably selected electrolyte additives can minimize dendritic growth when additive adsorption onto active hydrogen evolution sites occurs, reduce the solubility of ${\mathrm{Zn}(\mathrm{OH})}_{4}^{2-}$ in KOH solutions at concentrations lower than 6 M, increase passivation and internal resistance in the presence of induced ZnO precipitation, and decrease of hydrogen evolution by additive adsorption on electrochemically active sites [20]. The coatings can also be used to improve the cycle life of zinc electrodes or the zinc powders, e.g., a polyaniline coating [21]. Such coatings should ensure the migration of OH^−^ ions in order to facilitate the charging/discharging processes while inhibiting the migration rate of ${\mathrm{Zn}(\mathrm{OH})}_{4}^{2-}$ ions while discharging.

The MABs with alkaline electrolytes provide higher corrosion resistance of metals which ensures longer cycle life and higher activity of oxygen electrocatalysis in comparison with acidic and neutral electrolytes [6]. In the MABs with alkaline electrolytes, KOH, NaOH, and LiOH are the most commonly used. KOH electrolytes with high concentrations are characterized by the highest ionic conductivity and the lowest viscosity [6]. It was reported that carbon materials undergo corrosion at high oxidation potentials [22]. The use of carbon-based gas-diffusion layers cause electrode degradation, which leads to a shortened cycle life of the rechargeable Zn-air battery. Hence, carbon-free O_2_ cathodes for high-performance of the MABs are being sought. Metal-based gas-diffusion layers obtained in the form of porous Ni foam [23,24], stainless-steel mesh [22] and titanium mesh [25] using an appropriate surface modification, were proposed as media for gas diffusion in both aqueous and non-aqueous MABs. Solid and porous titanium has already been proposed in the literature for the production of metal-air batteries [25,26]. The electrical conductivity of titanium changes from 1.92 × 10^6^ S m^−1^ to 2.38 × 10^6^ S m^−1^ depending on the ASTM grade [27]. The proposal to use titanium results from the fact that the Ti electrode is more stable than carbon electrode, and after appropriate surface modification it possesses enhanced electrical conductivity. On the surface of titanium mesh, titanium nitride nanotube arrays were produced to which RuO_x_ nanoparticles were loaded as a catalyst without the need to use binders, which increased electrical conductivity [26].

Recent studies show that additive manufacturing, also known as three-dimensional (3D) printing, can be successfully used to produce porous metallic materials for electrochemical energy applications [28]. It is predicted that in the near future it will be possible to print not only components for the batteries, but also full 3D batteries. Among the available 3D printing methods such as fused deposition modeling (FDM), inkjet printing, stereolithography (SLA), and select laser melting (SLM), the latter method offers the possibility of producing complex geometric shapes based on a 3D CAD model. This allows precise control over not only the geometry of the product, but also shape, size, and distribution of the pores in the proposed structure. This creates promising perspectives for fast prototyping and low-cost manufacture of metallic porous materials which geometries will be precisely tailored to specific electrochemical requirements.

An important part of the whole process is the preparation of a 3D model of a porous structure, which will be produced. Typically, an elementary cell model is developed in CAD programs with the help of several basic objects (e.g., sphere, cylinder, cube) which are usually bounded by logical operations. Then, the elementary cell is reproduced in 3D space [29,30]. Recently much attention is devoted to the ability of generating cellular structures based on mathematical formulas with describe triply periodic minimal surfaces (TPMS) [31,32,33,34,35,36,37,38]. Minimal surfaces are defined as surfaces with zero mean curvature at all points. TPMS are minimal surfaces which are periodic in three independent directions [34]. They divide space into two intertwined labyrinth domains with smooth joints and curvatures. There are several ways for generating the coordinates of TPMS. The parametric TPMS representation are known as the Enneper–Weierstrass formula. It was shown that basing on this formula the exact computation of the Cartesian coordinates of the fundamental patch of several TPMS surfaces: Gyroid (G) [35], Diamond (D) [36], and Primitive (P) [37] was possible [35,36,37]. TPMS can be also approximated by the periodic nodal surface [38]. Based on simple trigonometric functions, with approximate TPMS, a mesh surface can be easily generated. The selection of the appropriate function and adjustment its parameters enable a control of the shape, size, distribution of pores and the total porosity of the cellular lattice model in a wide range [39].

In our previous studies, we showed that cellular lattice structures can be successfully manufactured using SLM process from titanium and titanium pre-alloyed powder [31,32,33]. The presented investigations focused on manufacturability of cellular lattices and the influence of unit cell geometry and relative density on the mechanical response of cellular lattice. In this study, we continue our interest in the commercially pure titanium Grade 2 (CpTi G2) cellular lattice with TPMS architecture. The main purpose of the undertaken research was to determine the corrosion resistance of three types of the TPMS: G, D, and I-2Y in 0.1 M KOH solution saturated with oxygen towards their potential use as media for gas diffusion in aqueous MABs. In order to understand the mechanism and kinetics of electrochemical corrosion of the CpTi G2 cellular lattices, a weakly concentrated 0.1 M KOH solution was selected. Our research on employing such 3D structured gas diffusion networks as an alternative to unstable carbon electrodes is preliminary. Research for the future applications of these materials on gas diffusion electrodes in high energy density metal-air batteries for the supply of oxygen requires the development of a method of surface modification of the obtained cellular structures with increased corrosion resistance. Hydrophobic binder materials to ensure the coexistence of gas and liquid phase in the pore network will also be under investigation.

## 2. Materials and Methods

### 2.1. Generation of TPMS Mesh Surface

**Figure 1:**
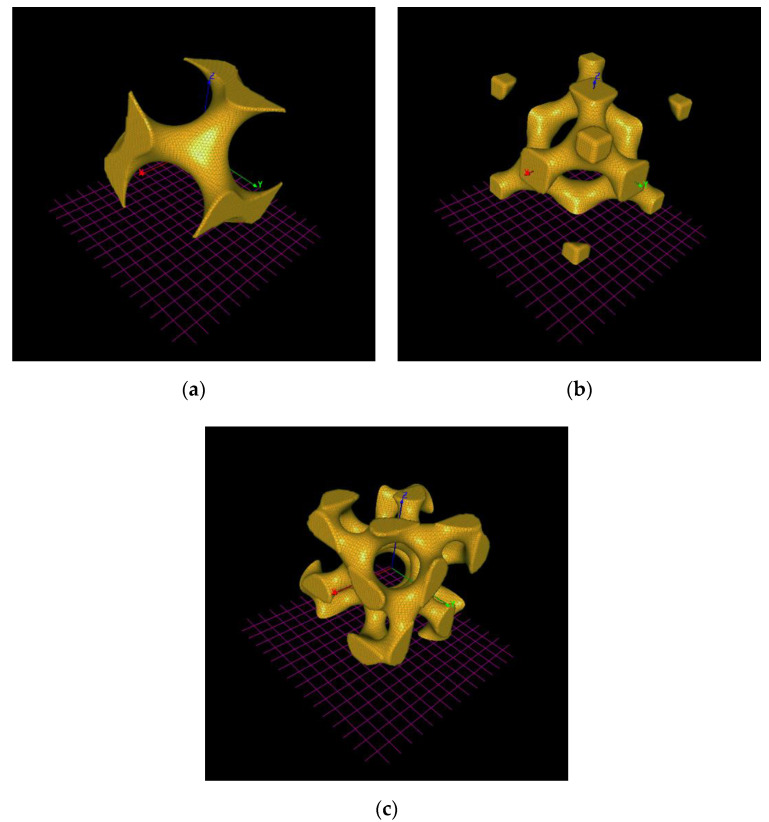
The triply periodic minimal surfaces (TPMS) basic unit cells libraries: (**a**) G; (**b**) D; (**c**) I-2Y.

The CpTi G2 cellular lattice with the TPMS architecture was modeled using the following types of the TPMS: G, D, and I-2Y [35,36,37,38]. The TPMS basic unit cells libraries are shown in Figure 1.

Periodic surfaces composed of simple trigonometric functions were used to approximate the selected TPMS:

*Φ*_G_(*x*, *y*, *z*) = *k*_1_·(cos(*x*)·sin(*y*) + cos(*y*)·sin(*z*) + cos(*z*)·sin(*x*)) − *k*_2_·(cos(2·*x*)·cos(2·*y*) + + cos(2·*z*)·cos(2·*x*)) − *k*_3_ (1)

*Φ*_D_(*x*, *y*, *z*) = *k*_1_·(sin(*x*)·sin(*y*)·sin(*z*) + sin(*x*)·cos(*y*)·cos(*z*) + cos(*x*)·sin(*y*)·cos(*z*) + + cos(*x*)·cos(*y*)·sin(*z*)) − *k*_2_·(cos(4·*x*) + cos(4·*y*) + cos(4·*z*)) − *k*_3_ (2)

*Φ*_I-2Y_(*x*, *y*, *z*) = −*k*_1_·(sin(2·*x*)·cos(*y*)·sin(*z*) + sin(2·*y*)·cos(*z*)·sin(*x*) + sin(2·*z*)·cos(*x*)·sin(*y*)) − *k*_2_·(cos(2·*x*)·cos(2·*y*) + cos(2·*y*)·cos(2·*z*) + cos(2·*z*)·cos(2·*x*)) − *k*_3_ (3)

For each type of the TPMS, model parameters such as *k*_1_, *k*_2,_ and *k*_3_ were chosen to prepare a structure featured with a porosity of *V*_v_ = 80%. The total porosity of the model (*V*_v_) stands for the ratio of the volume of pores (*V*_p_) to the total volume of the surrounding area of the model (*V*_tot_):

(4) $$V_{v}=\frac{V_{p}}{V_{\mathrm{tot}}}$$

**Figure 2:**
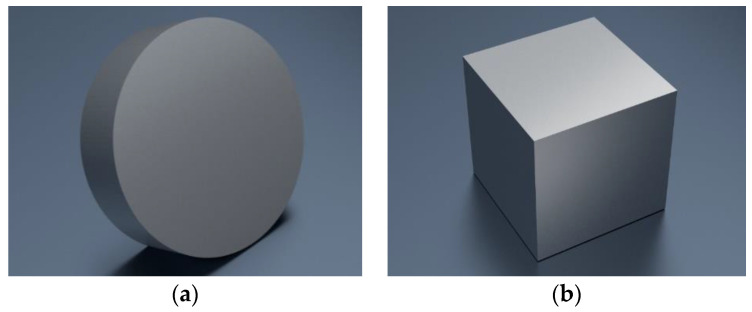
Full solids constituting a reference point for 3D modeling: (**a**) The cellular lattice with TPMS architecture; (**b**) Bulk specimen.

**Figure 3:**
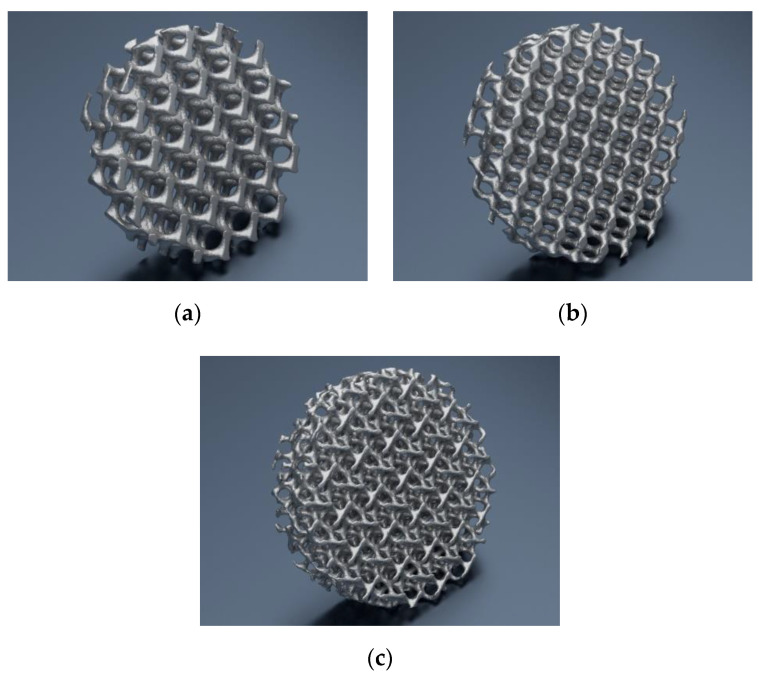
Visualization of the cellular lattice model with TPMS architecture: (**a**) G80; (**b**) D80; (**c**) I-2Y80.

It was assumed that the modeling area would be a disc with a diameter of ø15 mm and a height of 4 mm (Figure 2a). The full solid constituting a reference point for 3D modeling of bulk specimen was a cube with side length 10 mm (Figure 2b). The 3D mesh that was a set of coordinates of the vertices and the set of elements defining the topology of the mesh was exported to *.stl format. Figure 3 shows the finished models, while their basic parameters are presented in Table 1.

### 2.2. Selective Laser Melting of the Cellular Lattice Specimens

Cellular lattice specimens were obtained on the Renishaw AM250 SLM machine (Renishaw, Gloucestershire, UK) equipped with modulated pulse laser with a maximum power of 400 W and wavelength of 1064 nm. The commercially available CpTi G2 powder (EOS GmbH, Munich, Germany) with a chemical composition in accordance with ASTM F67 [40] was used. The surface morphology of the CpTi G2 powder and the cellular lattice specimens was investigated by scanning electron microscopy (SEM) using a JEOL JSM-6480 microscope (JEOL Ltd., Tokyo, Japan). An SEM image of the CpTi G2 powder (Figure S1) with its detailed characteristics and selective laser melting parameters (Table S1) can be found in the Supplementary Materials.

**Figure 4:**
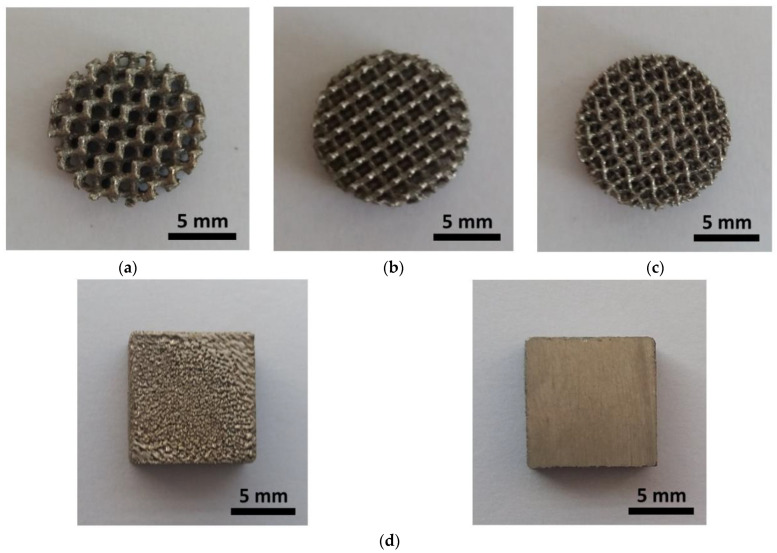
CpTi G2 produced by SLM: (**a**) G80 cellular lattice; (**b**) D80 cellular lattice; (**c**) I-2Y80 cellular lattice; (**d**) Bulk specimen in general view (left) and cross-sectional view (right).

After the SLM process, the cellular lattice specimens (Figure 4a–c) and comparative bulk specimen in the form of the cube, were cut from the base plate by wire electrical discharge machining. Plates 4 mm thick were cut from the cube for the corrosion tests (Figure 4d). Any trapped loose powder particles were removed from the obtained specimens by cleaning with a flow of compressed air and twice sonication for 30 min in ethanol.

SEM observations of the cellular lattice specimens and bulk CpTi obtained by SLM were carried out before and after corrosion test on an inclined table to reflect the 3D structure.

### 2.3. Topographic Studies

Surface topography maps of the investigated materials were registered using Scanning Electrochemical Workstation PAR Model 370 (Princeton Applied Research, Oak Ridge, USA) equipped with a tungsten Kelvin probe (KP, ø500 µm, Princeton Applied Research, Oak Ridge, TN, USA). The scanning area was 6 × 6 mm^2^ and the distance between the probe and the sample was ca. 100 µm. The quantitative analysis of surface heights distribution involved the analysis of local height magnitude in relation to the arithmetic average (*z*_av_), that was set to zero. To move the *z*_av_ to zero a constant was subtracted from each value on the topography map. The parameters that describe quantitatively the material surface topography, i.e., the arithmetic mean deviation (*S*_a_), maximum peak height (*S*_p_), maximum valley depth (*S*_v_), skewness (*S*_sk_), and excess kurtosis (*S*_ku_) were determined. The detailed description of the parameters can be found elsewhere [41,42,43,44,45,46].

### 2.4. Corrosion Resistance Studies

CpTi G2 cellular lattice with TPMS architecture of G80, D80, I-2Y80, and the bulk specimen manufactured by SLM were subjected to electrochemical measurements in 0.1 M KOH solution saturated with oxygen at 25 °C by means of the Autolab/PGSTAT30 computer-controlled electrochemical system (Metrohm Autolab B.V., Utrecht, The Netherlands). The working electrode (WE) was CpTi G2. The counter electrode (CE) was used in the form of a platinum foil with a surface area of 4 cm^2^. The Hg|HgO|0.1 M NaOH reference electrode (RE) was connected to the electrolyte using Luggin capillary filled with 0.1 M KOH solution. All the potentials are referred to the normal hydrogen electrode (NHE). The Nernst equation, *E* = *E*° − 0.059 pH, for the potential conversion from Hg|HgO|0.1 M NaOH electrode to NHE was used [47]. To normalize the measured currents to the current densities, the geometric surface area of the cellular structures was determined based on 3D models, which was approximated by a mesh of triangles. The geometric surface area of the cellular structures was calculated as the sum of the areas of all triangles that recreated a given surface (Parameter *S*, Table 1). A diagram of the electrochemical cell configuration (Figure S2) can be found in the Supplementary Materials.

The measurements of open circuit potential, *E*_OC_, were carried out for 2 h. Next, the Tafel curves in the range of potentials ±100 mV relative to the *E*_OC_ were recorded with the polarization rate of *v* = 1 mV s^−1^. The obtained polarization curves were the basis for the Tafel extrapolation method carried out in order to determine the corrosion resistance parameters such as corrosion potential (*E*_cor_), corrosion current density (*j*_cor_), anodic (*b*_a_) and cathodic (*b*_c_) Tafel slopes, polarization resistance (*R*_p_), and corrosion rate (*CR*) at the *E*_cor_ expressed in linear units.

The electrochemical impedance spectroscopy (EIS) measurements were conducted under potentiostatic control at the *E*_cor_ using the range of frequency (*f*) from 10^4^ to 10^−3^ Hz. The *f* resolution was 0.003%. Ten *f* per decade were used. The excitation signal was a sine wave with a small amplitude of 10 mV. In order to assess the validation of the raw EIS data, the Kramers–Kronig (K–K) relations were applied [48]. The analysis of the experimental EIS data was carried out using the selected equivalent electrical circuits. The complex non-linear least squares (CNLS) method with modulus weighting was used. The CNLS-fitting procedure was realized by the EQUIVCRT program [48,49]. The importance of the particular elements in the equivalent electrical circuits was checked using the statistical F test.

The anodic polarization curves were recorded from a potential 150 mV more negative in relation to the *E*_OC_ to 8.76 V vs. NHE with the polarization rate of *v* = 1 mV s^−1^.

All electrochemical measurements were repeated three times. The determined values of the corrosion resistance parameters were given as average values with their standard deviations.

## 3. Results and Discussion

### 3.1. Surface Morphology

**Figure 5:**
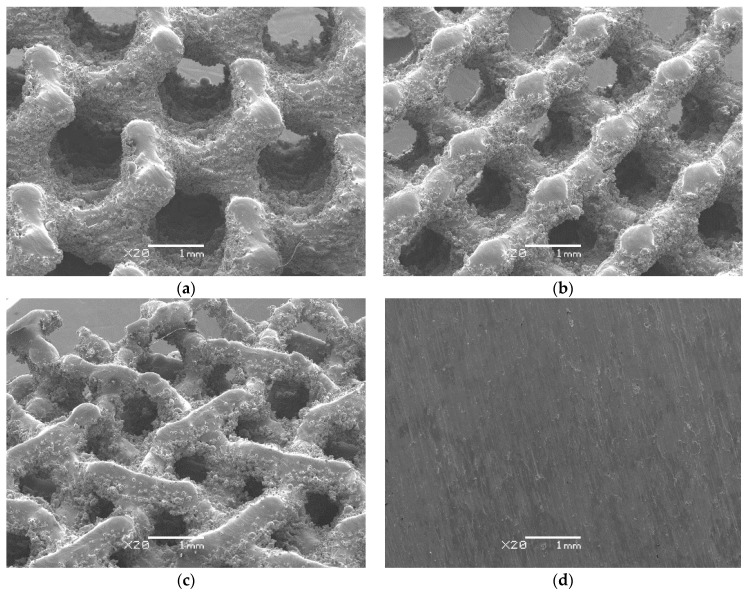
SEM image of surface morphology of the CpTi G2 produced by SLM: (**a**) G80 cellular lattice; (**b**) D80 cellular lattice; (**c**); I-2Y80 cellular lattice; (**d**) Bulk.

SEM observations reveal complex surface morphology of the CpTi G2 cellular lattice with the TPMS architecture (Figure 5a–c) in contrast to the undeveloped surface morphology of the bulk specimen (Figure 5d).

No loose powder residue that may have remained in the pores of the cellular lattice after the SLM process was observed. However, large amounts of partially melted powder particles or agglomerates were attached to the surface of the struts, which caused their surface to be rougher. This is particularly evident in the direction perpendicular to the construction direction and on sloping angle struts. This unfavorable phenomenon is commonly observed in the metallic structures produced by SLM method [31,32,33,50,51], and may have the effect of reducing corrosion resistance. These irregular surfaces can also act as stress concentration factors leading to a reduction in part strength. It was reported that the laser penetration and the infiltration effect had a strong influence on the surface roughness of the metallic specimens manufactured by SLM [50].

We can also observe the lack of delamination on the struts, which proves the correctness of the process of bonding individual layers in the SLM process. Moreover, the lower surfaces of the struts are rougher than the upper parts due to thermal diffusion. As a result of this phenomenon, caused by a large temperature difference between the sintered CpTi G2 and the loose powder particles surrounding it, the powder only partially melts and adheres to the struts. Part of the powder particles are melted by the laser beam carried to the powder surface along a pre-marked path, and next bonded to the boundary of each layer. The manufacturing of inclined struts is partly based on the previously sintered layer and the loose powder. Some of the powder particles beneath each layer may be fully or partially melted and then bonded to the lower material layer.

### 3.2. Surface Topography

**Figure 6:**
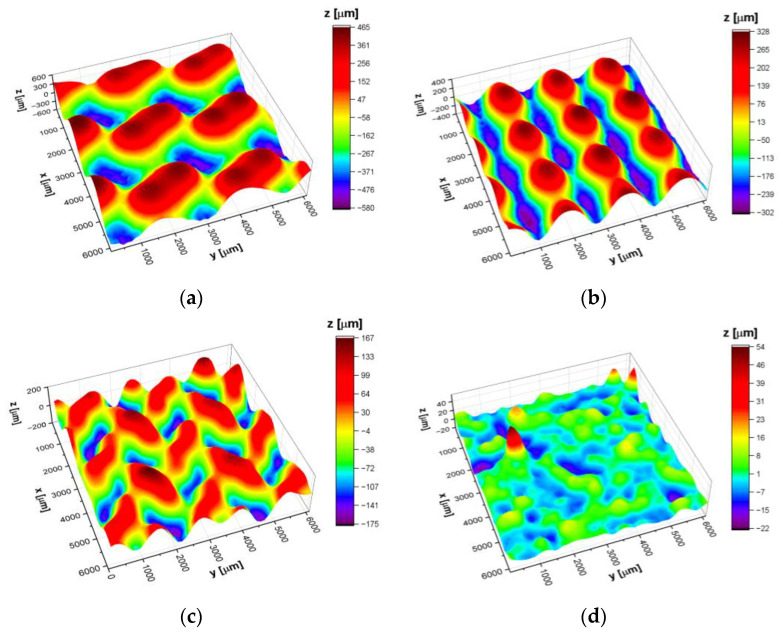
Topography maps determined for the CpTi G2 produced by SLM: (**a**) G80 cellular lattice; (**b**) D80 cellular lattice; (**c**) I-2Y80 cellular lattice; (**d**) Bulk.

Figure 6 shows surface topography maps of the investigated materials and corresponding statistical parameters are listed in Table 2.

Statistical analysis of the maps allows us to determine the arithmetic mean deviation (*S*_a_), maximum peak height (*S*_p_), and maximum valley depth (*S*_v_) which may be used to determine significant deviations in the surface texture. The values of *S*_a_, *S*_p_ and *S*_v_ parameters indicate increasing heights of peaks and valleys in the following order: bulk CpTi, I-2Y80 cellular lattice, D80 cellular lattice, and G80 cellular lattice. Note that the G80 specimen has the highest *S*_p_ and *S*_v_ values (8 and 25 times higher in comparison with bulk CpTi SLM) among all the investigated materials. Skewness (*S*_sk_) and excess kurtosis (*S*_ku_) describe the shape of the surface heights distribution and give information on the morphology of the surface texture. In particular, skewness describes the degree of symmetry of the surface heights about the average value i.e., when *S*_sk_ > 0 or *S*_sk_ < 0 then the predominance of peaks or valleys on the material surface is observed. *S*_ku_ > 0 indicates the presence of extremely high peaks/deep valleys while the *S*_ku_ < 0 is characteristic for periodically varying surface heights, i.e., for those surfaces in which privileged direction can be distinguished. It was found that for I-2Y80, D80, and G80 cellular lattices *S*_sk_ and *S*_ku_ parameters change in the range from −0.1 to +0.1 and from −0.8 to −1.2, thus, surface heights are symmetrically spread around the average value (surface is free of extreme peaks or valleys) and simultaneously surface heights follow a uniform distribution. Note that bulk CpTi SLM has a completely different surface texture. In the case of this specimen*,* *S*_sk_ and *S*_ku_ parameters indicate asymmetry of the height distribution and presence of extremely high peaks spread on the material surface.

### 3.3. Open Circuit Potential Studies

**Figure 7:**
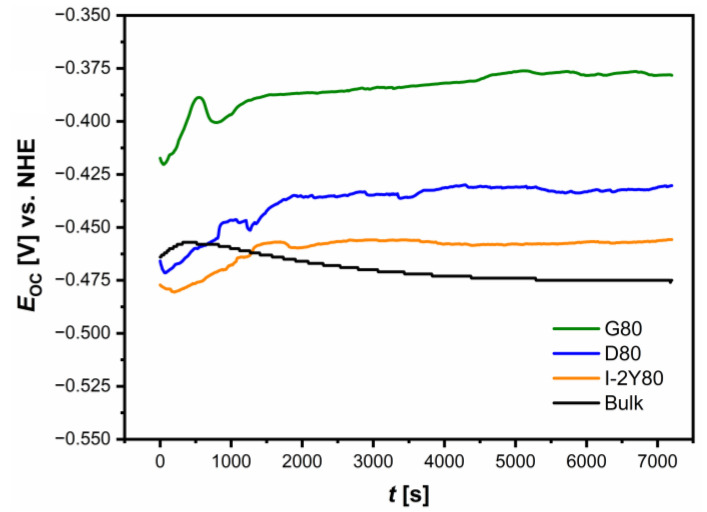
Dependence of open circuit potential on immersion time for the CpTi G2 cellular lattice with TPMS architecture of G80, D80, I-2Y80, and the bulk electrode in 0.1 M KOH solution saturated with O_2_ at 25 °C.

The *E*_OC_ parameter was used as a parameter determining the initial corrosion resistance of the CpTi G2 under study. Figure 7 presents the *E*_OC_ for cellular lattice with TPMS architecture and bulk electrode as a function of the immersion time (*t*) in 0.1 M KOH solution saturated with O_2_ at 25 °C.

The course of the *E*_OC_ = *f*(*t*) curves is mild. The ionic-electron equilibrium of the CpTi G2 electrode *|* 0.1 M KOH solution interface was established after about 5000 s. The stable *E*_OC_ value corresponds to the approximate *E*_cor_ value. For all the electrodes tested, a small maximum is visible at the initial stage of the test, followed by a slow stabilization of the *E*_OC_. Such nature of the changes suggests the presence of intact passive layers on the electrode surface. The lowest *E*_OC_ value of −0.475(9) V is observed for the bulk CpTi G2 electrode, which indicates the lowest corrosion resistance among all tested materials. After reaching the maximum in the initial stage of the study, the *E*_OC_ values slightly decrease with immersion time for this electrode, whereupon a nearly constant value of the potential is observed due to the stability of the passive layer. The reason for that probably can be the slow dissolution of the spontaneous oxide layer [52].

The measurements carried out in open loop of potentiostat for all the cellular structures with TPMS architecture studied reveal a slight increase in the *E*_OC_ values after recording the peak in the initial immersion stage. An increase in the *E*_OC_ towards positive values suggests the formation of protective passive layers on the surface of these electrodes or the sealing of the self-passive oxide layer. The average *E*_OC_ for all CpTi G2 cellular lattice electrodes is more positive in comparison with the *E*_OC_ for the bulk electrode ranging from −0.456(7) V for I-2Y80 cellular lattice electrode to −0.378(5) V for G80 cellular lattice electrode, respectively. The highest average *E*_OC_ value for CpTi G2 cellular lattice with TPMS architecture of G80 type indicates the presence of passive oxide layer with the strongest barrier properties.

### 3.4. Tafel Curves

**Figure 8:**
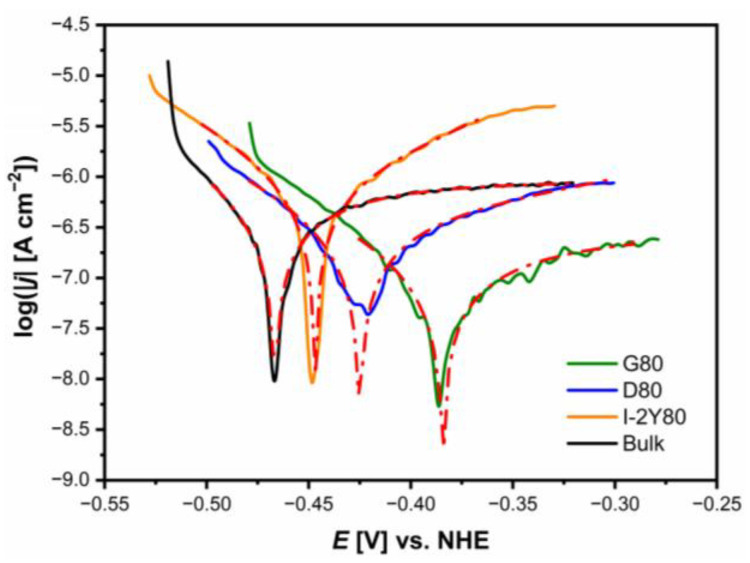
Tafel curves for the CpTi G2 cellular lattice with TPMS architecture of G80, D80, I-2Y80, and the bulk electrode in 0.1 M KOH solution saturated with O_2_ at 25 °C. Continuous lines represent the experimental data and red dash dotted lines are the fitting results obtained using the Tafel extrapolation.

The Tafel curves for the CpTi G2 cellular lattice with TPMS architecture of G80, D80, I-2Y80, and in comparison with the bulk electrode, recorded in 0.1 M KOH solution saturated with O_2_ at 25 °C, are presented in Figure 8. Based on these results, corrosion resistance parameters were determined by the Tafel extrapolation method (Table 3).

The lowest average *E*_cor_ value of −0.467(2) V is determined for the bulk CpTi G2 electrode. Similar value of the *E*_cor_ was reported for the CpTi G2 plates in alkaline solutions [53]. Figure 8 shows the shift of the average *E*_cor_ towards the anodic potentials for all cellular lattice with TPMS architecture compared to the bulk electrode. This electrochemical behavior demonstrates an improvement in the corrosion resistance of the cellular lattice tested. The highest average *E*_cor_ of −0.384(8) V is observed for CpTi G2 cellular lattice with TPMS architecture of G80 type (Table 3). This result can be correlated with the type of TPMS architecture. For G80 cellular lattice the largest changes in the surface topography were visible (Figure 6, Table 2).

The average *j*_cor_ values for all CpTi G2 cellular lattice with TPMS architecture and bulk electrode are on the order of 10^−7^ A cm^−2^. These *j*_cor_ values are typical for titanium in low concentrated aqueous solutions [52,53]. The highest average *j*_cor_ of 8.96(5) × 10^−7^ A cm^−2^ is observed for the CpTi G2 bulk electrode (Table 3). In the case of all cellular structures, there is a decrease in *j*_cor_, the strongest in the case of the G80 electrode, more than 3 times in relation to the bulk electrode. The *j*_cor_ parameter is directly proportional to the dissolution rate of the passive oxide layers, however, it cannot be used as a kinetic parameter for comparison of the corrosion resistance of the tested materials.

The *b*_c_ and *b*_a_ Tafel slopes were determined based on the dependencies given in [54]. The obtained *b*_c_ values for all tested electrodes are lower as compared to the *b*_a_ values (Table 3). These results indicate similar mechanism of corrosion for the CpTi G2 cellular lattice with TPMS architecture of G80, D80, I-2Y80, and the bulk electrode, in which anodic processes are faster than the cathodic reactions.

The CpTi G2 in alkaline solution feature passive behavior like any metal (Me) covered with a layer of its oxide. The passive oxide layer is a physical barrier limiting both anode and cathode processes. At the *E*_cor_, the self-passive TiO_2_ layer on the electrode surface is oxidized very slowly. Under aerobic conditions, oxygen acts as the oxidation agent. The following reaction takes place in which metal oxides and hydroxides or hydrated oxides are formed, but without the hydrogen formation [55]:

(5) $$\mathrm{Me}+n\cdot H_{2}O+n/2\cdot O_{2}↔{\mathrm{Me}(\mathrm{OH})}_{n}$$

The pH of solution plays an important role in corrosion process. However, as a consequence of the autoprotolysis equilibrium of water:

(6) $$H_{2}O↔H^{+}+{\mathrm{OH}}^{-}$$

the basic Reaction (5) remains valid for alkaline and acidic solutions.

The average value of *R*_p_ = 3.88 × 10^4^ Ω cm^2^ is the lowest for the bulk CpTi G2 electrode (Table 3). For the cellular structures, an increase in the *R*_p_ parameter is observed, which has the greatest value for the G80 cellular structure.

The values of the *CR* at the *E*_cor_ change inversely with the *R*_p_ (Table 3). All the tested electrodes are characterized by material consumption of 10^−3^ mm yr^−1^. However, all cellular structures corrode slower compared to the bulk electrode, which is of great importance from the application point of view. The CpTi G2 cellular lattice with TPMS architecture of G80 reveals the lowest value of *CR*, which is over 3 times lower as compared to the bulk CpTi G2.

### 3.5. Electrochemical Impedance Spectroscopy

**Figure 9:**
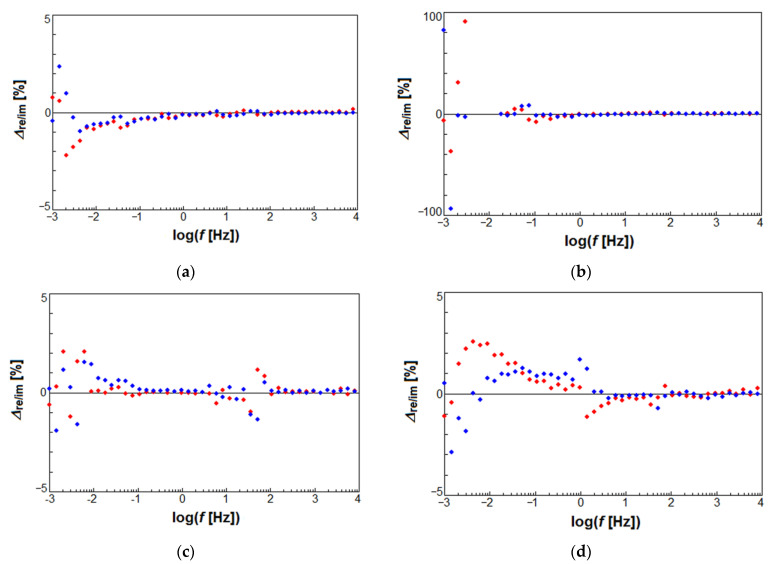
The K–K test residuals for a K–K test of the experimental EIS data obtained at the *E*_cor_ for the CpTi G2 in 0.1 M KOH solution saturated with O_2_ at 25 °C: (**a**) G80 cellular lattice; (**b**) D80 cellular lattice; (**c**) I-2Y80 cellular lattice; (**d**) Bulk. The real and imaginary part differences are represented by red and blue dots, respectively.

The validity of raw EIS data for the CpTi G2 in 0.1 M KOH solution saturated with O_2_ at 25 °C was confirmed by the K–K test [48,56]. In Figure 9, relative differences Δ_re,i_ and Δ_im,i_ between the experimental EIS data and the fit using the K–K test as a function of the frequency log did not exceed 3% in the entire range of tested frequencies. Only for D80 cellular lattice in Figure 9b, a few points with large deviations at the lowest frequencies were observed, which were not K–K transformable. These points were not included in the CNLS-fit of EIS spectra. According to the literature, the possible reasons for large deviations at the lowest frequencies in Figure 9b can be surface microscopic roughness or porosity, surface heterogeneities and geometry-induced nonuniform distribution of current and potential [56]. It was confirmed that the EIS spectra met the K–K relation proving the correctness of the experimental data.

**Figure 10:**
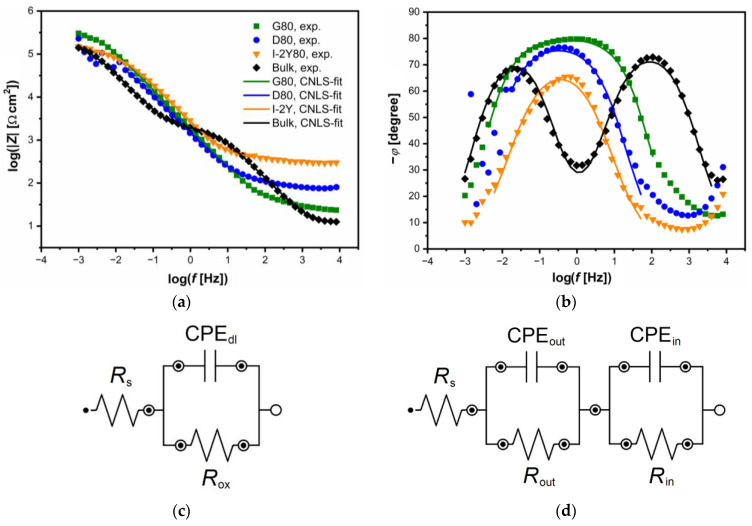
Bode diagrams for the CpTi G2 cellular lattice with TPMS architecture of G80, D80, I-2Y80, and the bulk electrode in 0.1 M KOH solution saturated with O_2_ at 25 °C with the equivalent electrical circuit models used for CNLS-fitting: (**a**) Magnitude; (**b**) Phase angle; (**c**) One-CPE model; (**d**) Two-CPE model. Experimental data (symbols) and continuous lines (CNLS fit).

Figure 10 shows the experimental Bode diagrams (symbols). In Figure 10a,b the same symbols are used. The Bode diagrams showing the dependence of log |*Z*| on log *f* have a slope in the range of medium frequencies of about −1 (Figure 10a). The value of log |*Z*| at *f* = 1 mHz is higher for all cellular lattice tested as compared to the bulk electrode which proves their higher corrosion resistance. The highest value of log |*Z*|*_f_*_= 1 mHz_ of 5.4(4) Ω cm^2^ is observed for G80 cellular lattice. Figure 10b presents corresponding Bode diagrams in the form of phase angle (*φ*) in dependence on log *f*. A plateau can be observed in the medium frequency range for G80 cellular lattice confirming strong passive behavior. In all cases, the maximum values of *φ* is below −80°. One time constant is visible in the electrical circuit for all cellular lattices. Such impedance behavior is typical for metallic electrodes covered with oxide layer [52,56]. For the bulk electrode, two time constants are present in the electrical circuit. This result is consistent with the literature data for CpTi G2 plates in alkaline solutions [53]. The obtained high values of |*Z*|*_f_*_→0_ (Figure 10a) and *φ* (Figure 10b) indicate that the tested materials are characterized by capacitive behavior and high corrosion resistance.

The EIS results with respect to the barrier properties of the passive oxide layers on the surface of CpTi G2 cellular lattice were approximated using the equivalent electrical circuit model shown in Figure 10c. This is the so-called one-CPE model with four adjustable parameters: *R*_s_, *T*_dl_, *ϕ*_dl_, and *R*_ox_, and displays one semicircle on the complex-plane plot [52,56]. In this model, *R*_s_ is the solution resistance, *R*_ox_ is the charge transfer resistance through the TiO_2_|electrolyte interface, and the CPE_dl_ is the constant phase element (CPE) which is a nonlinear element introduced instead of a capacitor corresponding to the electrical double layer capacitance. The impedance definition of the CPE (${\hat{Z}}_{\mathrm{CPE}}$) is given by the following equation:

(7) $${\hat{Z}}_{\mathrm{CPE}}=\frac{1}{T{\left(j\omega \right)}^{\phi }}$$

where *T* is the capacitance parameter of the CPE in F cm^−2^ s *^ϕ^*^−1^, and *ϕ* is a CPE exponent related to the constant phase angle, *α* = 90°(1−*ϕ*). The *ϕ* parameter is dimensionless and takes values ≤ 1 [56].

The EIS results obtained for the bulk CpTi electrode were approximated using the equivalent electrical circuit model shown in Figure 10d. This is the so-called two-CPE model with seven adjustable parameters: *R*_s_, *T*_out_, *ϕ*_out_, *R*_out,_ *T*_in_, *ϕ*_in_, *R*_in_ [53,56]. This model assumes a two-layer structure of the passive oxide film on the surface of the titanium electrode and displays two semicircles on the complex-plane plot. In this model, the semicircle at high frequencies is attributed to the presence of an outer oxide layer with a porous structure (*R*_s_, *T*_out_, *ϕ*_out_, *R*_out_). The semicircle at low frequencies is associated with the presence of an inner oxide layer directly adjacent to the substrate, which exhibits strong barrier properties (*T*_in_, *ϕ*_in_, *R*_in_).

The CNLS-fitted data using the electrical equivalent circuits presented in Figure 10c,d are continuous lines in Figure 10a,b, respectively. We can see an exceptionally good quality of CNLS-fitting. All parameters obtained using the equivalent electrical circuit model shown in Figure 10c to approximate the experimental EIS data for the CpTi G2 cellular lattices with TPMS architecture are listed in Table 4. Table 5 presents all parameters as the fitting result obtained using the equivalent electrical circuit model shown in Figure 10d to approximate the experimental EIS data for the bulk CpTi electrode in Figure 10b.

The highest value of *R*_ox_ = 3.21(6) × 10^5^ Ω cm^2^ is observed for G80 cellular lattice, which is in accordance with the *R*_p_ parameter determined in the potentiodynamic measurements (Table 3). For the cellular structure D80 and I-2Y80, the *R*_ox_ values are of the same order as *R*_p_ (Table 3). This proves the correctness of the conducted impedance measurements. The physico-chemical meaning of the *R*_ox_ parameter is associated with the corrosion process described by the Equation (5). In the case of the bulk CpTi electrode, the charge transfer resistance related to outer oxide layer takes a value two orders smaller as compared to *R*_in_ of 2.13(6) × 10^5^ Ω cm^2^, which testifies to stronger barrier properties of the inner oxide layer thermodynamically more stable (Table 5).

The *ϕ*_dl_, *ϕ*_out_, and *ϕ*_in_ parameters are significantly deviated from 1 (Table 4 and Table 5). This is due to the presence of physical, chemical or geometrical inhomogeneities [56]. Taking into account the surface topology of the CpTi G2 electrodes, the origin of the CPE dispersion can be associated with the surface roughness.

The high corrosion resistance of CpTi G2 can be attributed to its surface oxide layer. Before starting the corrosion tests, an oxide layer was formed spontaneously on the surface of both the bulk CpTi G2 electrode and the cellular lattices. From a thermodynamic point of view, Ti can react quickly with oxygen and stable Ti oxides are formed [57]. The initial layer formed in the air consists mainly of amorphous TiO_2_ with a band gap energy of 3.05 eV, which may be influenced to some extent by the surroundings.

### 3.6. Anodic Polarization Curves

**Figure 11:**
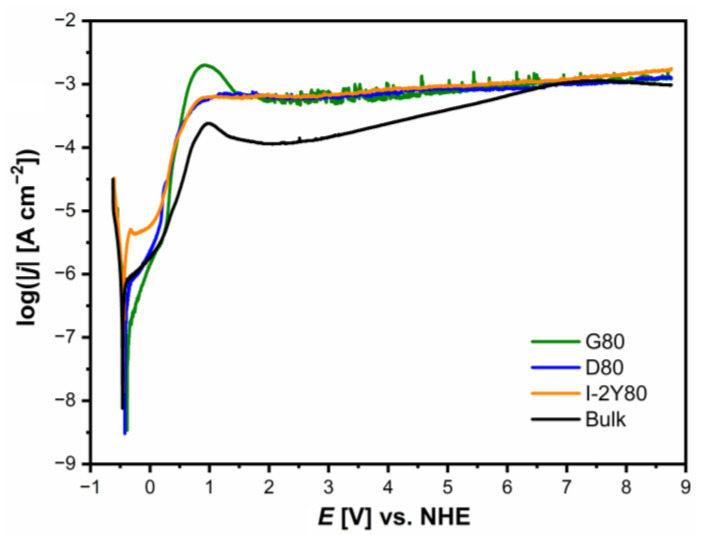
Anodic polarization curves for the CpTi G2 cellular lattice with TPMS architecture of G80, D80, I-2Y80, and the bulk electrode in 0.1 M KOH solution saturated with O_2_ at 25 °C.

The analysis of the anodic polarization curves presented in semi-log form in Figure 11 shows a similar course for all tested materials. A shift towards anodic potentials for CpTi G2 cellular lattice with TPMS architecture of G80, D80, and I-2Y80 as compared to the bulk electrode is observed due to increase in the corrosion resistance. For potentials with values lower than *E*_cor_, the tested electrodes show resistance to corrosion. With potential values above *E*_cor_, the oxidation process begins according to Equation (5). On all the anodic polarization curves, a narrow passive range exists in which the oxide layers exhibit protective properties. The lowest passive current densities of the order of 10^−6^ A cm^−2^ are observed for the G80 cellular lattice. The passive range ends with a potential of about 0.2 V. After exceeding the passive range, an increase in the current densities with the increase of the anodic potential is observed until the maximum at about 1 V in the transpassive range is reached. The increase in anodic current density is related to the oxidation of titanium cations that formed the passive layers. This process leads to the formation of transpassive layers containing titanium in a higher oxidation state or to the complete dissolution of the passive layers. The transpassive layers exhibit barrier properties up to 8.76 V, but the protection of the titanium substrate is provided with higher current densities of the order of 10^−3^ A cm^−2^ compared to the passive current density values. It is worth noting that the transpassive layers on the surface of cellular structures show greater stability compared to the self-passive oxide layer on the surface of the bulk electrode.

The assessment of the corrosion damage of the tested materials after recording the anodic polarization curves was carried out based on SEM observations. SEM image of surface morphology of the CpTi G 2 after potentiodynamic test (Figure S3) can be found in the Supplementary Materials. After highly destructive corrosion tests, no differences in the surface morphology of the tested materials were observed compared to the initial state (Figure 5), which proved the excellent corrosion resistance of the tested CpTi G2 in an alkaline environment.

## 4. Conclusions

SLM technology was successfully used to produce CpTi G2 cellular lattices with a controlled total porosity of 80%. Under proposed operating parameters 3D cellular lattice structures with the TPMS architecture of G80, D80, and I-2Y80, were obtained. SEM examination of the CpTi G2 cellular lattices with the TPMS architecture revealed much more complex surface morphology compared to the bulk CpTi SLM. A statistical analysis of the surface topography maps allowed us to determine significant deviations in the surface texture of the obtained materials. It was found that for I-2Y80, D80, and G80 cellular lattices surface heights were symmetrically spread around the average value, and the surface heights were evenly distributed. In the case of the bulk CpTi SLM, the determined statistical parameters indicated the asymmetry of the height distribution and the presence of extremely high peaks distributed over the specimen surface.

The effect of the sample geometry on corrosion resistance of the CpTi G2 was determined in 0.1 M KOH solution saturated with oxygen at 25 °C using the open circuit potential method, Tafel curves, anodic polarization curves, and EIS. The corrosion resistance of the CpTi G2 cellular lattice with TPMS architecture determined based on direct current measurements was higher in comparison with the bulk CpTi G2 SLM as evidenced by higher values of *E*_OC_, *E*_cor_, *R*_p_, and lower values of *j*_cor_, *CR* at *E*_cor_. For all tested materials, the results of the Tafel extrapolation method indicated similar mechanism of corrosion with faster anodic processes than cathodic reactions, and oxygen acting as the oxidation agent. Anodic polarization curves revealed passive-transpassive behavior for the CpTi G2 cellular lattices and comparative CpTi G2 SLM.

The experimental EIS results with respect to the barrier properties of the passive oxide layers on the surface of CpTi G2 can be approximated using the one-CPE model for the cellular lattices with TPMS architecture, and two-CPE model for the bulk electrode. Based on alternating current measurements, the capacitive behavior of all materials was found. The enhanced corrosion resistance featured the 3D cellular lattice structures.

Among all the materials tested, the highest corrosion resistance was revealed for the CpTi G 2 cellular lattice with TPMS architecture of G80. The proposed cellular structures manufactured by SLM can be proposed as promising materials with increased corrosion resistance elongating the life cycle for gas diffusion in alkaline MABs.

## Figures and Tables

**Table 1:** The basic parameters of the real cellular lattice with TPMS architecture, where: *S*—geometric surface area, *V*—volume of the model, *V*_v_—total porosity of the model, *S*/*V*—surface area to volume ratio.

Type of the TPMS	*S* [cm^2^]	*V* [cm^3^]	*V*_v_ [%]	*S*/*V* [cm^2^ cm^−3^]
G80	7.99	0.14	81.2	57.0
D80	10.03	0.15	79.8	66.9
I-2Y80	13.46	0.13	82.5	103.5

**Table 2:** Statistical parameters obtained for the topography maps of the CpTi G2 produced by SLM; *S*_a_ is the arithmetic mean deviation, *S*_p_ is the maximum peak height, *S*_v_ is the maximum valley depth, *S*_sk_ is the skewness, and *S*_ku_ is the excess kurtosis.

CpTi G2	*S*_a_ [μm]	*S*_p_ [μm]	*S*_v_ [μm]	*S*_sk_	*S*_ku_
G80 cellular lattice	232.2	463.2	577.3	−0.08	−1.19
D80 cellular lattice	145.8	327.3	301.1	0.10	−1.22
I-2Y80 cellular lattice	59.7	166.2	174.9	−0.11	−0.81
Bulk	4.8	53.9	22.4	1.44	8.09

**Table 3:** Corrosion resistance parameters determined by the Tafel extrapolation method for the CpTi G2 cellular lattice with TPMS architecture of G80, D80, I-2Y80, and the bulk electrode in 0.1 M KOH solution saturated with O_2_ at 25 °C (see Figure 8).

CpTi G2	*E*_cor_ [V]	*j*_cor_ [A cm^−^^2^]	*b*_c_ [V dec^−^^1^]	*b*_a_ [V dec^−^^1^]	*R*_p_ [Ω cm^2^]	*CR* at *E*_cor_ [mm yr^−1^]
G80 cellular lattice	−0.384(8)	2.85(14) × 10^−7^	0.153(10)	0.291(6)	1.53 × 10^5^	2.48 × 10^−3^
D80 cellular lattice	−0.425(9)	3.58(39) × 10^−7^	0.108(10)	0.286(32)	9.51 × 10^4^	3.11 × 10^−3^
I-2Y cellular lattice	−0.447(3)	7.75(25) × 10^−7^	0.094(2)	0.277(2)	3.93 × 10^4^	6.74 × 10^−3^
Bulk	−0.467(2)	8.96(5) × 10^−7^	0.107(1)	0.317(11)	3.88 × 10^4^	7.79 × 10^−3^

**Table 4:** The parameters with their standard deviations obtained using the equivalent electrical circuit model for corrosion shown in Figure 10c to approximate the experimental EIS data for the CpTi G2 cellular lattices with TPMS architecture in 0.1 M KOH solution saturated with O_2_ at 25 °C. *R*_s_ was approximately equal to 11 Ω cm^2^ for all measurements.

CpTi G2	*T*_dl_ [F cm^−2^ s *^ϕ^^−^*^1^]	*ϕ*_dl_	*R*_ox_ [Ω cm^2^]
G80 cellular lattice	2.61(8) × 10^−6^	0.896(1)	3.21(6) × 10^5^
D80 cellular lattice	1.09(8) × 10^−6^	0.872(3)	1.41(9) × 10^5^
I-2Y80 cellular lattice	2.18(2) × 10^−4^	0.809(5)	3.03(7) × 10^4^

**Table 5:** The parameters with their standard deviations obtained using the equivalent electrical circuit model for corrosion shown in Figure 10d to approximate the experimental electrochemical impedance spectroscopy (EIS) data for the bulk CpTi G2 electrode in 0.1 M KOH solution saturated with O_2_ at 25 °C. *R*_s_ was approximately equal to 11 Ω cm^2^.

CpTi G2	*T*_out_ [F cm^−2^ s *^ϕ^*^−1^]	*ϕ*_out_	*R*_out_ [Ω cm^2^]	*T*_in_ [F cm^−2^ s *^ϕ^*^−1^]	*ϕ*_in_	*R*_in_ [Ω cm^2^]
Bulk	2.34(7) × 10^−5^	0.877(5)	2.15(4) × 10^3^	2.75(4) × 10^−4^	0.893(6)	2.13(6) × 10^5^

## Data Availability

The data presented in this study are available on request from the corresponding author.

## Supplementary Materials

The following are available online at https://www.mdpi.com/1996-1944/14/1/81/s1, Table S1: Selective laser melting parameters, Figure S1: SEM image of the CpTi G2 powder, Figure S2: Diagram of the electrochemical cell configuration, Figure S3: SEM image of surface morphology of the CpTi grade 2 after potentiodynamic test.
