# Supplementary material for: Corrosion Resistance of the CpTi G2 Cellular Lattice with TPMS Architecture for Gas Diffusion Electrodes
Source: Materials (Basel). 2020 Dec 26;14(1):81. doi: 10.3390/ma14010081 (PMC7795527; doi:10.3390/ma14010081)
Supplement: Supplementary file 1 [file materials-14-00081-s001.pdf]

materials

Supplementary Materials

Corrosion resistance of the CpTi G2 cellular lattice with TPMS architecture for gas diffusion electrodes

Bożena Łosiewicz*, Joanna Maszybrocka, Julian Kubisztal, Grzegorz Skrabalak and Andrzej Stwora

SEM image of the CpTi G2 powder

Figure S1 depicts the SEM image of the surface morphology of the CpTi G2 powder used for selective laser melting of the cellular lattice specimens with the triply periodic minimal surfaces architecture of G80, D80, I-2Y80, and comparative bulk specimen. The powder particles were characterized by an almost spherical shape and a smooth surface morphology. The diameter of the powder particles, determined on the basis of microscopic observations, ranged from 5 to 45 μm.

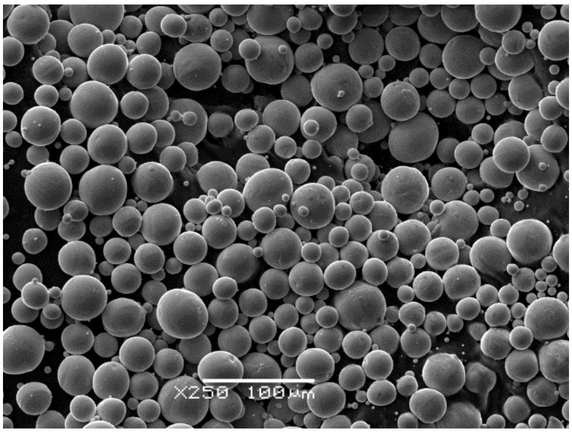

Figure S1. SEM image of the CpTi G2 powder.

Selective laser melting parameters

Table S1 presents the operating parameters of the selective laser melting process used to obtain the CpTi G2 cellular lattice specimens with the triply periodic minimal surfaces architecture of G80, D80, I-2Y80, and comparative bulk specimen. The selective laser melting process was carried out under an inert argon atmosphere with the oxygen content limited to 500 ppm.

Table S1. Selective laser melting parameters.

Operating parameter	Value
Laser power	300 W
Scan speed	300 mm s^{-1}
Point distance	65 μm
Exposure time	65 ms
Layer thickness	50 μm
Scan strategy	Meander

Diagram of the electrochemical cell configuration

Figure S2 shows a diagram of the electrochemical cell configuration used in electrochemical measurements for the CpTi G2 cellular lattice electrodes with the triply periodic minimal surfaces architecture of G80, D80, I-2Y80, and comparative bulk electrode, carried out in 0.1 M KOH solution saturated with oxygen at 25 °C. Corrosion resistance measurements were carried out in a classical three-electrode electrochemical cell with a non-separated space. The electrolyte volume was 80 ml. The WE and CE electrodes were parallely positioned face-to-face in the electrochemical cell. A diagram of the electrochemical cell configuration presents schematically the alignment and distance between the electrodes used.

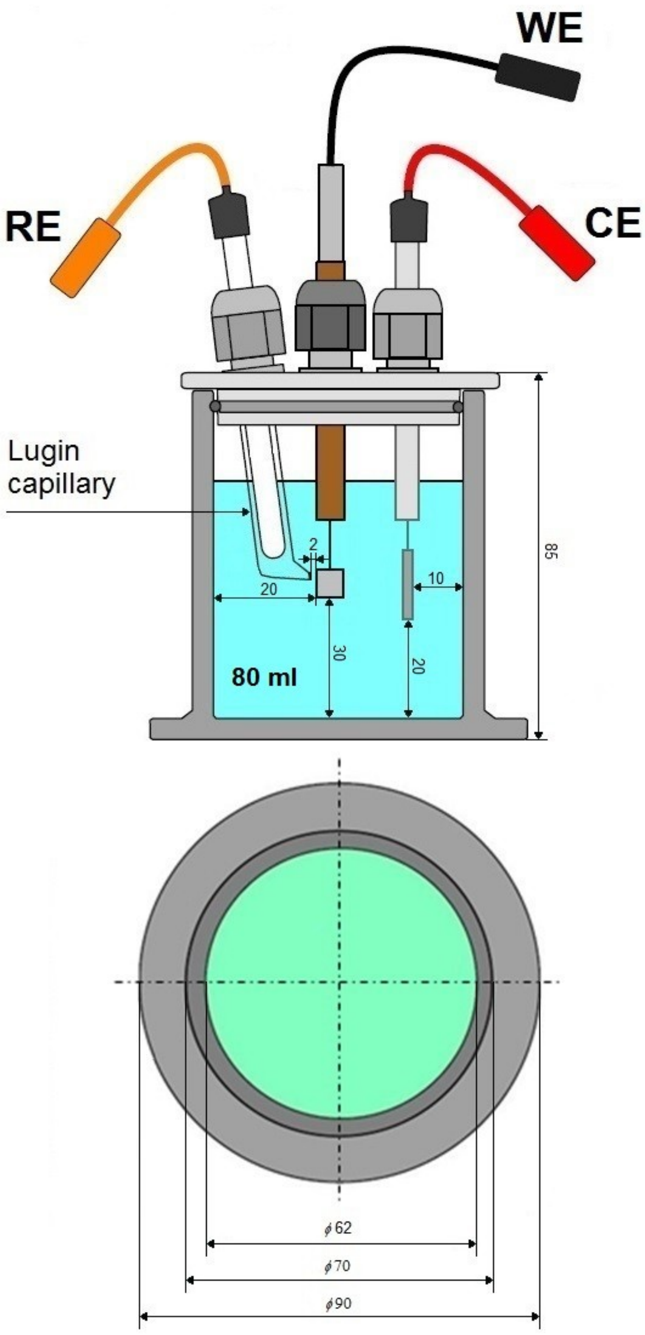

Figure S2. Diagram of the electrochemical cell configuration, where: WE – working electrode (CpTi G2), CE – counter electrode (Pt), and RE – reference electrode (Hg|HgO|0.1 M NaOH). Dimensions are given in mm.

SEM image of surface morphology of the CpTi G 2 after potentiodynamic test

Figure S3 depicts the SEM image of surface morphology of the CpTi G2 cellular lattice electrodes with the triply periodic minimal surfaces architecture of G80, D80, I-2Y80, and comparative bulk electrode, after potentiodynamic test. The SEM images of the surface morphology of the tested materials still reveal a rough surface, despite the potentiodynamic measurements used with a very high anodic potential limit. There is no smoothing of the surface morphology as a result of anodic dissolution. This means that the anodic dissolution of the passive oxide layers on the surface of CpTi G 2 is very slow. The obtained cellular structures with the triply periodic minimal surfaces architecture of G80, D80, I-2Y80 manufactured by the selective laser melting process can be proposed as promising electrode materials with increased corrosion resistance for gas diffusion in the alkaline metal-air batteries.

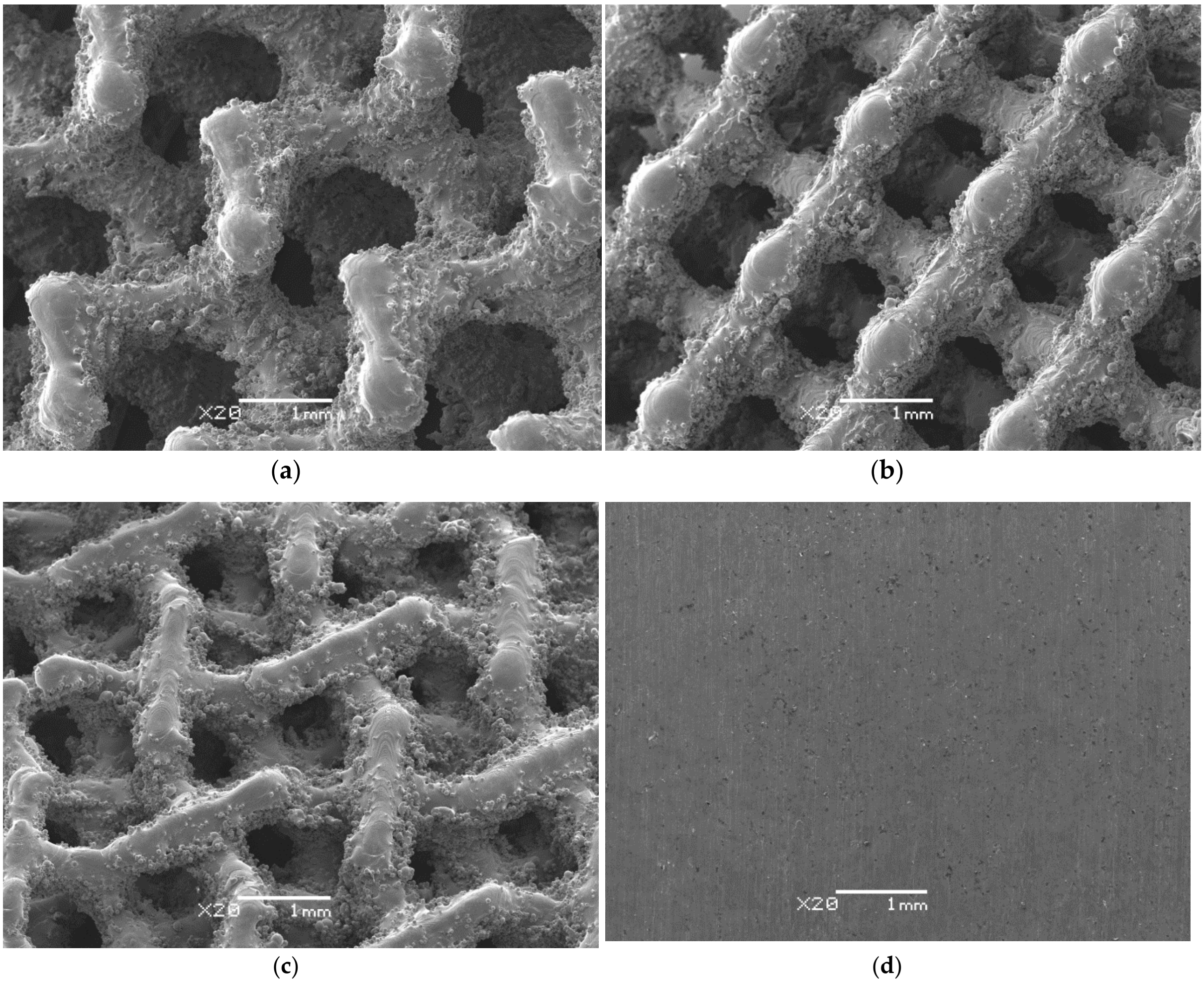

(**a**) (**b**)

(**c**) (**d**)

Figure S3. SEM image of surface morphology of the CpTi G 2 after potentiodynamic test: (**a**) G80 cellular lattice; (**b**) D80 cellular lattice; (**c**) I-2Y80 cellular lattice; (**d**) Bulk.
